# Advancing psychiatry trainee wellbeing and safety: Building on RANZCP Position Statement 48

**DOI:** 10.1177/10398562231211135

**Published:** 2023-10-31

**Authors:** Michael James Weightman, Andrew Amos, Edward Miller

**Affiliations:** Discipline of Psychiatry, 1066 The University of Adelaide, Adelaide, SA, Australia; Division of Tropical Health and Medicine, College of Medicine and Dentistry, 104397 James Cook University, Townsville, QLD, Australia; Division of Psychological Medicine, Faculty of Medical & Health Sciences, 1415 The University of Auckland, Auckland, New Zealand

**Keywords:** wellbeing, trainee, psychiatry, medical education

## Abstract

**Objective:**

The RANZCP recently released Position Statement 48 on the ‘safety and wellbeing of psychiatrists and those in psychiatry training’. This article will examine the five key domains highlighted by this statement and provide suggestions on how this guidance might relate to trainees. The domains covered are (i) safe workplaces free from discrimination, bullying, harassment, and violence; (ii) positive team cultures; (iii) positive professional peer relationships; (iv) supportive supervision and mentorship; and (v) work–life balance.

**Conclusions:**

In the context of the significant and complex demands of psychiatry training, Position Statement 48 helps to provide a framework for trainees and the people and systems that support them to understand, anticipate, and successfully manage the potential risks to trainee wellbeing and safety.

In February 2023, the Royal Australian and New Zealand College of Psychiatrists (RANZCP) released Position Statement 48 on the ‘safety and wellbeing of psychiatrists and those in psychiatry training’.^1^ This is important and timely given the increased demands on trainees in recent years,^2^ including working on the frontline through the COVID-19 pandemic; dealing with chronic understaffing in many health services; and high-profile impacts on progression to Fellowship due to uncertain exam delivery and changes to assessment requirements. In addition to facing many of the same stressors as psychiatrists, trainees must balance providing clinical care to their patients with the competing demands of training and assessment. This dual agency dilemma can be challenging to navigate, especially since services are becoming more reliant on trainees for regular service provision and supervision can be insufficient.^2^ Trainees must additionally negotiate the unusual pressures associated with the accumulation of responsibility from being a junior member of the multidisciplinary team at the beginning of training to becoming the senior decision-maker as consultant.

As a result, it is imperative that trainees develop robust wellbeing habits during their journey to Fellowship to avoid burnout and sustain professional practice as a consultant. Moreover, trainees who are safe and well are better placed to deliver high-quality care to the patients and communities they serve, whilst also experiencing increased career satisfaction and improved retention within the profession.^1–3^ Position Statement 48 lists five priorities that could assist all RANZCP members to establish and maintain safe and healthy workplace habits (Table 1). As part of this training-themed issue, the present article will expand on the ways in which the recommendations within the position statement could apply to trainees.

**Table 1.** Key domains within RANZCP Position Statement 48

1. Safe workplaces free from discrimination, bullying, harassment, and violence.
2. Positive team cultures.
3. Positive professional peer relationships.
4. Supportive supervision and mentorship.
5. Work–life balance.

## Safe workplaces

While safety should be a basic expectation of all workplaces, in mental healthcare the combination of high levels of distress in patients and carers; a workforce and support structure under significant dynamic stress; and caring for patient populations with a comparatively high prevalence of challenging behaviours or substance misuse can constitute formidable obstacles. As it may not be possible to fully address risks on a local level, it is crucial that trainees can escalate to external bodies when required to ensure their concerns are addressed.^4^ In particular, trainees should be aware of how to contact trainee representatives on their local Branch Training Committee (BTC). Unsafe work environments risk loss of accreditation: the RANZCP Committee for Training, in conjunction with the investigating BTC, can disaccredit an individual rotation or even a training network.^5^ This prospect of losing accreditation is a strong motivator for employers to address issues, as trainees are essential to maintain a safe level of clinical service.

From an industrial point of view, unions and the national medical associations of each country are also important resources.^4^ These organisations are focussed on protecting the rights and interests of their members as enshrined in their enterprise bargaining agreement and helping to resolve conflicts with employers. These organisations can also advocate for future improvements to working conditions or health systems. Notifying and logging instances of discrimination is important for building an accurate representation of workplace culture, which can provide evidence on which to base successful change.

## Positive team cultures

Creating a positive team culture for trainees is crucial for their professional development and wellbeing.^6^ However, the need to rotate through new workplaces every 6–12 months can make it difficult for trainees to successfully integrate into existing cultures. Challenges may include the limited time for relationship building; lack of continuity with reduced sense of belonging; and need to swiftly upskill into the training context of a new rotation. As with many training experiences, rapid accommodation to new workplace cultures can be extremely rewarding where well-prepared and supported trainees consider success is in their own control, or extremely stressful where the demands appear to overwhelm trainee perception of their own resources.

Employers can improve workplace culture by providing a welcoming and informative orientation process to every new trainee. Positive first impressions establish expectations and inculcate a supportive working environment. It is important to promote clear and open communication within clinical teams that encourages trainees to express their ideas, concerns, or questions without fear of judgement. Supportive supervision arrangements and regular team meetings also enhance effective communication and culture. Trainees should be provided regular constructive feedback, which helps improve skills and builds confidence. Finally, the efforts and achievements of trainees should be acknowledged and appreciated. Team culture is formed in the pursuit of shared goals by all members over time, shaped by the leadership of senior staff. Trainees working in teams with high leadership turnover (e.g. multiple locum doctors or ‘acting’ leadership representatives) could also report this during rotation feedback processes, or directly to their local BTC or union in serious cases.

Trainees also have opportunity to positively impact on team culture, as every new rotation allows the trainee to bring a fresh perspective and contribute their own ideas and insights to their new workplace. Maintaining an open and curious interpersonal approach can assist the trainee to learn about their new workplace and build professional relationships with their new colleagues. Active participation in unit meetings and social events such as team dinners increases the sense of belonging and helps humanise the workplace experience.

## Positive professional peer relationships

Trainees need positive relationships across multiple complementary networks: horizontal peer networks of fellow trainees; vertical peer networks of supervisors or senior registrars; and external peer networks including friends and family.^7^ Horizontal peer relationships can provide a valuable support network in which trainees can share experiences, seek advice, and receive emotional support from colleagues who understand the demands of their training. Vertical networks of senior colleagues allow opportunity to discuss complex cases and explore different perspectives on patient care. Senior colleagues can offer valuable experience, share resources, and provide feedback, which can all contribute to professional growth and enhanced clinical skills. Finally, it is important to have relationships outside of psychiatry to provide perspective, social connection, and assist with work–life balance. Nurturing these different relationships throughout training can have long-lasting benefits for trainees’ personal and professional growth long into their careers.

## Supportive supervision and mentorship

Trainee access to experienced supervisors provides guidance, support, and constructive feedback.^8^ All trainees must have an allocated supervisor for each rotation with protected weekly times for individual supervision. A shared plan around the aims and goals of supervision across the training rotation is essential, and best documented at the beginning of the working relationship. A regular time for supervision should be agreed upon and booked in a shared calendar. The focus of supervision should extend beyond simple clinical guidance and documenting workplace-based assessments to include overall professional growth and personal support.

Support should also be accessible outside formal supervision, especially given the additional assessor role that supervisors must now perform within the competency-based Fellowship framework. For example, collective reflection on clinical practice through Balint groups can benefit trainees,^9^ while formal mentorship arrangements with senior clinicians can help contain junior trainees’ anxiety as they begin their training journey.^10^ Personal individual psychotherapy is no longer a mandated general training requirement, but remains available and is arguably underutilised outside of advanced training in psychodynamic psychotherapy.^11^

## Work–life balance

The emotionally demanding and challenging nature of their work can place psychiatry trainees at potential risk with their own mental health. When surveyed, over a third of RANZCP members (including both trainees and fellows) endorsed either ‘often’ or ‘usually’ poor work–life balance.^3^ The modal contributing factor was ‘too much work to do in a limited time’, although paperwork and intrusion of work on family life were also commonly cited.^3^ A healthy work–life balance protects against burnout.^6^ Establishing this balance during training is essential, as adopting healthy routines and boundaries early on sets up good habits that can be maintained throughout one’s career. It is also important for supervisors to model such practices in their own work.

In an increasingly demanding workplace context, trainees are expected to learn and keep up with the psychiatric literature, evolving nosology, and treatment advances. Maximising learning efficiency can help maintain work–life balance. Useful learning tools include educational podcasts, concise literature reviews, and instructional articles. *Australasian Psychiatry* contains an extensive back catalogue of accessible articles pertinent to trainees on topics such as the centralised assessments, clinical skills, and wellbeing.^12^ The trainee-led podcast, *The Thought Broadcast*, provides high-yield training-specific content.^13^ Structured trainee study groups formed well in advance of examinations can both reduce stress and provide a mutually supportive forum for learning. Such groups help maintain perspective and emotional wellbeing, as well as increasing assessment preparedness.

## Limitations

There are inherent limitations to the impact of a position statement, particularly given that trainees are neither employed nor trained by the RANZCP itself. Rather, this is usually the purview of local public health services and Formal Education Courses run by BTCs or universities. The RANZCP therefore has minimal control over many of the factors discussed in this article and has limited powers to intervene beyond disaccrediting training posts. Secondly, the position statement overlooks the primary avenue by which the RANZCP could directly improve trainee wellbeing – rationalising the extensive programme of assessments. As examinations are a major source of distress for trainees,^3^ aiming for the minimum level of assessment to effectively detect competency could significantly improve wellbeing.

## Conclusion

RANZCP’s Position Statement 48 is an important and timely document focused on the wellbeing and safety of psychiatrists and psychiatrists in training. It helps to provide a framework for trainees and the people and systems that support them to understand, anticipate, and successfully manage the potential risks to trainee wellbeing while developing sustainable habits for long-term career satisfaction.
